# Associations between media use, self-efficacy, and health literacy among Chinese rural and urban elderly: A moderated mediation model

**DOI:** 10.3389/fpubh.2023.1104904

**Published:** 2023-03-09

**Authors:** Yebo Yu, Yibo Wu, Zhen Huang, Xinying Sun

**Affiliations:** ^1^Department of Social Medicine and Health Education, School of Public Health, Peking University, Beijing, China; ^2^Health Culture Research Center of Shaanxi, Key Research Base of Philosophy and Social Sciences in Shaanxi Province, Xi'an, Shaanxi, China

**Keywords:** media use, health literacy, self-efficacy, urban-rural residents, Chinese elderly

## Abstract

**Objectives:**

The influence of media use on health literacy among urban and rural elderly has been unknown in China. This study aims to examine the association between media use and health literacy and to explore the mediating role of self-efficacy and the moderating role of urban-rural residency.

**Methods:**

Based on the cross-sectional study of the Psychology and Behavior Investigation of Chinese Residents (PBICR) in 2022, a total of 4,070 Chinese old people aged 60 years and above were included. We adopted the simplified New General Self-Efficacy Scale (NGSES) and the simplified Health Literacy Scale-Short Form (HLS-SF) to measure self-efficacy and health literacy. Media use was measured using a self-administrated questionnaire.

**Results:**

Results showed that Chinese urban elderly had a higher frequency of media use than rural ones in the aspects of social activities, self-presentation, social action, leisure and entertainment, information acquisition, and business transactions through media (*P* < 0.001). Among all participants, self-presentation (*B* = 0.217, 95% CI: 0.040, 0.394), leisure and entertainment (*B* = 0.345, 95% CI: 0.189, 0.502), and information acquisition (*B* = 0.918, 95% CI: 0.761, 1.076) were significantly associated with health literacy. Self-efficacy partially mediated the effect of media use on health literacy (B_indirect_ = 0.045, 95% CI: 0.032, 0.058), accounting for 18.37% of the total effect. Urban-rural residency (*B* = 0.049, 95% CI: 0.024, 0.075) moderated the relationship between media use and self-efficacy significantly.

**Conclusion:**

The urban-rural gap in health literacy requires more attention. The promotion of media use and self-efficacy may play a role in eliminating health disparities.

**Limitations:**

As a cross-sectional study, it could not establish cause-effect relationships.

## 1. Introduction

In recent years, the rapid growth of informatical technology has brought about wide popularity of media use, not only traditional media, such as broadcast and newspaper, but also social media. Social media means the internet-based channels of mass personal communication (1), which allows the creation and exchange of user-generated content (2). In 2021, over 4.26 billion people were using social media across the world, and the number of media users is expected to reach 6 billion in 2027 (3). People take advantage of media functions to enrich their lives, such as communicating with friends and browsing the latest news (4).

Although some obstacles exist for old people to learn and use media, the trends of media usage among the elderly seem to be increasing worldwide with a growing aging population (5). A survey showed that 40% of Amerian aged 65 and older used social media in 2019 (6). In China, 119 million internet users aged 60 and older in 2021, with an internet penetration rate of 43.2% (7). This high rate indicates the popularity of media use among Chinese older adults. Nevertheless, as a huge economic gap between urban and rural areas in China, the media use of elderly living in cities and the countryside may show different conditions.

Health literacy is defined as the ability to obtain, process, understand, and communicate health-related information, which helps to make health decisions and to manage health status (8, 9). According to Sørensen et al. (10), health literacy included three domains: health care (access medical information or clinical issues), disease prevention (learn and protect against health risk factors), and health promotion (access information on determinants of health for regularizing healthy behaviors). Previous studies suggested that health literacy played an essential role in preventing and managing chronic diseases (11, 12). It's suggested that health literacy inequality was attributable to income inequalities (13), and inadequate health literacy was associated with a low socioeconomic position (14). Some studies reported that Chinese older adults have a relatively low level of health literacy (15), especially those living in rural areas (16). Therefore, it's necessary to investigate the urban-rural gap in health literacy and to explore potential factors influencing health literacy, for urban and rural elderly, respectively.

The process of media use is filled with information generating, exchanging, and assimilating. The users of media could obtain health-related information or skills through the process of media use. Therefore, it's reasonable to assume that the frequency of media use is associated with levels of health literacy (17). But whether the relationship above is positive or negative, experts haven't reached a uniform idea. Some studies found that the usage of media, such as television, the internet, and smartphones, is beneficial for the improvement of health literacy (18, 19). While some scholars hold the opinion that it was difficult to evaluate the authenticity of health-related information *via* media channels (20), which may play a negative impact on health literacy. As to the difference between urban and rural residents, the two groups may adopt diverse media use behaviors that exert different effects on health literacy. Pitifully, little literature has discussed this issue. As a result, it's worthwhile to test the influence of different types of media use on health literacy among Chinese older adults, stratified for urban and rural residents.

Self-efficacy is one's belief in their ability to execute activities that will access satisfactory outcomes (21). Self-efficacy could be regarded as an important indicator that evaluates people's confidence, which has been widely used as a mediator in health promotion programs (22). Previous literature has found an association between media use and self-efficacy (23, 24). And several studies have supported the effect of self-efficacy on health-related knowledge and behaviors (25). In this research, self-efficacy may mediate the relationship between media use and health literacy. On the one hand, the usage of media is a novel thing for Chinese old adults, as they were born and grew up in an era with low economy and technology, and most of them have low education levels. Generally, learning media use is a challenge for the Chinese elderly (26). Once they can use the functions of the media, such as chatting with friends living far away, their self-efficacy would increase. On another hand, people with high self-efficacy could grasp useful health-related skills and distinguish between true and false information, which promotes the improvement of their health literacy (27). Hence, it's suggested that self-efficacy is a potential mediator of the association between media use and health literacy.

Moreover, urban-rural residency may have the potential to moderate the association between media use and self-efficacy. There are gaps between urban and rural areas in China in aspects of income levels and entertainment facilities (28). Compared with old adults living in the countryside, seniors living in cities have more chances to contact and make use of media. For example, the service of buying medicine online through applications on smartphones exists in most cities in China (29). However, this service is rarely available in rural areas of China. Under this circumstance, urban old people could obtain more achievement from media use, which further improves their self-efficacy. In addition, the acquaintances of the urban elderly are also likely to use media in their daily life, which could give urban elderly more positive feedback and guidance on media use (30). Consequently, urban old people are supposed to have a higher level of self-efficacy than rural aged, with the same frequency of media use, which could lead to greater urban-rural health inequalities.

**Figure 1:**
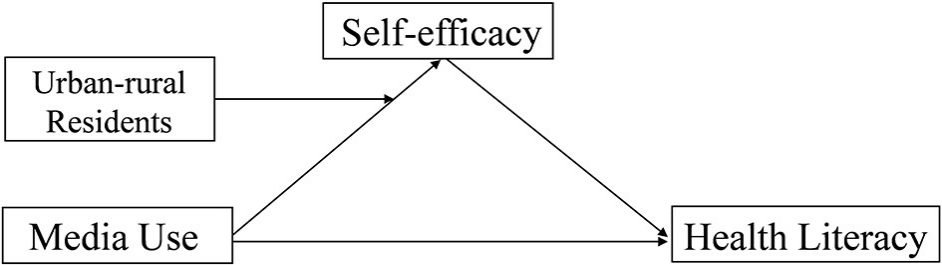
Conceptual framework.

So far, very little literature has discussed the influence of different types of media use on health literacy for urban and rural older adults, or taken self-efficacy and urban-rural residency into consideration. In light of the above concerns, four hypotheses would like to be proposed for Chinese old people (Figure 1).

Hypothesis 1: There are differences between urban and rural elderly in terms of media use and health literacy.

Hypothesis 2: The health literacy of urban and rural elderly is associated with different types of media use.

Hypothesis 3: Self-efficacy will mediate the association between media use and health literacy.

Hypothesis 4: Urban-rural residency will moderate the effect of media use on self-efficacy. This means that urban elderly have a stronger relationship between media use and self-efficacy than rural aged.

## 2. Materials and methods

### 2.1. Study design

The cross-sectional study, the Psychology and Behavior Investigation of Chinese Residents (PBICR) (31), was conducted in China between 20th June and 31st August 2022. Stratified sampling and quota sampling were adopted. A total of 148 cities were included, from the 23 provinces, 5 autonomous regions, and 4 municipalities directly under the central government (Beijing, Tianjin, Shanghai, and Chongqing). Electronic questionnaires were distributed and collected through face-to-face or real-time video guidance by investigators. Informed consent was obtained before participants were voluntarily involved in our research, which contained the study's purpose, anonymity, confidentiality, and other related rights. Excluding ineligible respondents whose surveys had extremely short completion times or inconsistent answer patterns, 21,916 samples entered our research. We selected 4,070 old people aged 60 years old or above. The study was approved by the Ethics Committee of the Health Culture Research Center of Shaanxi (JKWH-2022-02).

### 2.2. Measurement

#### 2.2.1. Media use

After reviewing related literature systematically (32, 33), media use was measured by a self-administrated questionnaire. Six items correspond to six categories of media use behaviors, including social communication (e.g., communicating with others), self-presentation (e.g., regarding the virtual community as a self-display platform, recording daily life and sharing personal feelings on it), social action (e.g., defending their rights or standing up for the justice of others), leisure and entertainment (e.g., playing games, listening to music or watching short videos), information acquisition (e.g., browsing the news and searching for various information), and business transactions (e.g., online shopping or online payment) *via* media. All items were rated on a five-point Likert scale (1 = never use, 5 = always use). The total score ranges from 6 to 30 and a higher score indicates more frequent usage of media. The Cronbach's alpha was 0.870 in this study.

#### 2.2.2. Self-efficacy

New General Self-Efficacy Scale (NGSES) was developed by Chen, Gully, and Eden (34). Self-efficacy in this study was measured using a simplified version of NGSES. With two random datasets including 11,031 participants, classical test theory and Mokken model of item response theory were used to simplify NGSES from 8 items to 3 items, which showed high reliability and validity. The three items (“When facing difficult tasks, I am certain that I will accomplish them,” “I will be able to successfully overcome many challenges,” and “I am confident that I can perform effectively on many different tasks”) rating with five responses (1 = very disagree, 5 = very agree). The total score was the sum of the scores of all three items, and a higher score means a higher level of self-efficacy. The Cronbach's alpha was 0.912 in this study.

#### 2.2.3. Health literacy

Health Literacy Scale-Short Form (HLS-SF) was developed by Duong et al. (9) and has shown high reliability and validity (35). We used the simplified version of HLS-SF to measure health literacy in this study. A total of 7,449 participants were included and two datasets were generated randomly for the simplified process of HLS-SF. Based on classical test theory, a 9-item version of the scale (HLS-SF 9) was obtained through simplification, which has high reliability and validity for Chinese residents (36). The total scale includes three domains (health care, disease prevention, and health promotion), and each dimension was measured by three items. Each item was rated with four responses (1 = very difficult, 4 = very easy). Accordingly, the higher the scores, the greater level of health literacy that the participants own. The total score was calculated by summing all items. The Cronbach's alpha was 0.923.

#### 2.2.4. Moderating and confounding variables

As the potential factor moderating the association between media use and self-efficacy, urban-rural residency refers to living in an urban or rural area in the last 3 months.

Potential confounders included age, gender (male/female), ethnicity (Han nationality/else), education (primary school or below/middle school/undergraduate or above), occupation status (retired/on a job/ else), per capita monthly household income (3,000 and below/3,001–5,000/5,001–9,000/9,001 and above, yuan/RMB), marital status (married/else), living alone (yes/no), chronic disease (yes/no), smoking habit (never smoking/used smoking/smoking nowadays), and drinking habit (never drinking/used drinking/drinking nowadays).

### 2.3. Statistical analysis

Descriptive analysis was used to describe the background characteristics of the samples. Mann-Whitney U test was adopted to test the difference in 11 items of media use and health literacy between urban and rural residents. After applying Bonferroni correction, here we choose *P* < 0.0045 (0.05/11) as the significance level to protect findings from type-I errors (37). In the subsequent statistical analyses, the significance levels were set at *P* < 0.05. We used multivariable linear regression models to detect the association between media use behaviors and health literacy stratified for urban-rural residency. Mediating and moderating effects were tested through PROCESS 4.1 invented by Hayes (38). Model 4 of PROCESS was used to explore the mediating role of self-efficacy on the association between media use and health literacy, adjusted for age, gender, residency, ethnicity, education level, current marital status, monthly personal income, occupational status, living alone, chronic disease, smoking, and alcohol. Control for the same variables except for residency, model 7 of PROCESS was used to test the moderating role of urban-rural residents on the relationship between media use and self-efficacy in the moderated mediation model. Then we included three aspects of health literacy (health care, disease prevention, and health promotion) into the moderated mediation model separately. The number of bootstrap samples was 5,000 in this study. All the above statistical analysis processes were implemented through SPSS version 26.0.

## 3. Results

### 3.1. Descriptive analysis

The mean age of participants was 68.77 years old. 50.2% of them were males, and 90.7% of them were of Han nationality. Urban residents occupied 55.9% and rural residents accounted for 44.1%. Most participants have married (83.3%). 47.8% of them had primary school or below education level, 43.9% had 3,000 yuan or below income per month, and 55.3% were retired. The proportion of living alone was 14.7%. Some of them reported chronic disease (57.9%), smoking (17.5%), and drinking (14.2%) currently. The means (SD) scores of total media use, self-efficacy, and health literacy were 24.00 (9.6), 15.00 (10.5), and 36.00 (25.1), respectively (Table 1).

**Table 1:** Descriptive analysis of background characteristics (*N* = 4,070).

	**N/mean**	**%/SD**
Age (mean, SD)	68.77	6.3
**Gender**		
Male	2,044	50.2
Female	2,026	49.8
**Residency in the last 3 months**		
Urban	2,276	55.9
Rural	1,794	44.1
**Ethnicity**		
Han nationality	3,693	90.7
Else	377	9.3
**The highest educational level attained**		
Primary school or below	1,946	47.8
Middle school	1,787	43.9
Undergraduate or above	337	8.3
**Current marital status**		
Married	3,389	83.3
Else	681	16.7
**Monthly personal income (¥)**		
3,000 and below	1,786	43.9
3,001–5,000	1,295	31.8
5,001–9,000	723	17.8
9,001 and above	266	6.5
**Occupational status**		
Retired	2,251	55.3
On a job	569	14.0
Else	1,250	30.7
**Living alone**		
No	3,472	85.3
Yes	598	14.7
**Chronic disease**		
No	1,715	42.1
Yes	2,355	57.9
**Smoking**		
Now	713	17.5
Ever	261	6.4
Never	3,096	76.1
**Alcohol**		
Now	579	14.2
Ever	570	14.0
Never	2,921	71.8
The total score of media use (mean, SD)	24.00	9.6
The score of self-efficacy (mean, SD)	15.00	10.5
The score of health literacy (mean, SD)	36.00	25.1

SD, standard deviation.

Table 2 shows that urban elderly had higher scores of all types of media use than rural old people (*P* < 0.001), including social communication, self-presentation, social action, leisure and entertainment, information acquisition, business transactions, and total media use. Urban-aged people also had higher scores in health literacy, including health care, disease prevention, health promotion, and total health literacy, compared with rural old ones (*P* < 0.001).

**Table 2:** The difference in media use and health literacy between urban (*N* = 2,276) and rural (*N* = 1,794) elderly in China.

		**Mean ±SD**	***Z***	***P*** **-value**
**Media use**				
Social communication	Urban	2.14 ± 1.07	−5.886	< 0.001
	Rural	1.91 ± 1.15		
Self-presentation	Urban	1.41 ± 1.26	−5.645	<0.001
Rural	1.18 ± 1.19		
Social action	Urban	1.52 ± 1.22	−6.241	<0.001
	Rural	1.29 ± 1.23		
Leisure and entertainment	Urban	1.75 ± 1.25	−5.835	<0.001
	Rural	1.52 ± 1.25		
Information acquisition	Urban	1.97 ± 1.18	−9.672	<0.001
	Rural	1.59 ± 1.23		
Business transactions	Urban	1.46 ± 1.27	−6.186	<0.001
	Rural	1.22 ± 1.25		
Total score of media use	Urban	10.25 ± 5.57	−8.547	<0.001
	Rural	8.70 ± 5.76		
**Health literacy**				
Health care	Urban	8.77 ± 1.89	−13.883	<0.001
	Rural	7.93 ± 1.82		
Disease prevention	Urban	8.91 ± 1.82	−14.472	<0.001
	Rural	8.05 ± 1.84		
Health promotion	Urban	8.52 ± 2.01	−14.094	<0.001
	Rural	7.62 ± 1.99		
Total score of health literacy	Urban	26.20 ± 5.24	−15.192	<0.001
	Rural	23.59 ± 5.09		

SD, standard deviation. Mann-Whitney U test was used. The significance level was set at *P* < 0.0045 after applying Bonferroni correction.

### 3.2. Multiple linear regression analysis

The relationships between media use and health literacy, stratified for urban-rural residency, are presented in Table 3. Among all participants, self-presentation (*B* = 0.217, 95% CI: 0.040, 0.394), leisure and entertainment (*B* = 0.345, 95% CI: 0.189, 0.502), and information acquisition (*B* = 0.918, 95% CI: 0.761, 1.076) were significantly associated with health literacy. For urban old people, health literacy was positively related to leisure and entertainment (*B* = 0.295, 95% CI: 0.084, 0.506) and information acquisition (*B* = 0.780, 95% CI: 0.561, 0.999). For rural elderly, their health literacy had positive associations with self-presentation (*B* = 0.334, 95% CI: 0.059, 0.609), leisure and entertainment (*B* = 0.435, 95% CI:0.206, 0.664), and information acquisition (*B* = 0.989, 95% CI: 0.762, 1.216), and had a negative correlation with social communication (*B* = −0.247, 95% CI: −0.475, −0.019). With health literacy as independent variables and media use as dependent variables, shown as Supplementary Table 1, the associations between health literacy and media use were positively significant, both among urban and rural old people.

**Table 3:** Multiple linear regression analyses for the relations of six types of media use to health literacy.

	**Model 1: all participants**		**Model 2: urban elderly**		**Model 3: rural elderly**	
	***B*** **(95% CI)**	β	***B*** **(95% CI)**	β	***B*** **(95% CI)**	β
Social communication	−0.111 (−0.272, 0.049)	−0.023	0.064 (−0.159, 0.288)	0.013	−0.247 (−0.475, −0.019)	−0.056^*^
Self-presentation	0.217 (0.040, 0.394)	0.050^**^	0.115 (−0.115, 0.346)	0.028	0.334 (0.059, 0.609)	0.078^*^
Social action	0.098 (−0.067, 0.263)	0.023	0.165 (−0.057, 0.387)	0.038	0.028 (−0.218, 0.274)	0.007
Leisure and entertainment	0.345 (0.189, 0.502)	0.081^***^	0.295 (0.084, 0.506)	0.070^**^	0.435 (0.206, 0.664)	0.107^***^
Information acquisition	0.918 (0.761, 1.076)	0.210^***^	0.780 (0.561, 0.999)	0.176^***^	0.989 (0.762, 1.216)	0.240^***^
Business transactions	−0.015 (−0.186, 0.155)	−0.004	0.065 (−0.159, 00.288)	0.016	−0.121 (−0.385, 0.142)	−0.030

*B*, non-standardized coefficients; β, Beta, standardized coefficients; CI, confidence interval.

* *p* < 0.05;

** *p* < 0.01;

*** *p* < 0.001.

Multiple linear regressions were adopted, with health literacy as dependent variables, and six types of media use as independent variables. All models were adjusted for age, gender, residency, ethnicity, education level, current marital status, monthly personal income, occupational status, living alone, chronic disease, smoking, and alcohol.

### 3.3. Mediation model

Table 4 shows that the path coefficients of “media use→ self-efficacy” (*B* = 0.053, 95% CI: 0.040, 0.067) and “self-efficacy→ health literacy” (*B* = 0.842, 95% CI: 0.784, 0.899) and “media use→ health literacy” (*B* = 0.200, 95% CI: 0.175, 0.226) were all significant. Self-efficacy partially mediated the effect of media use on health literacy, with an effect value of 0.045, accounting for 18.37% of the total effect (Table 5).

**Table 4:** Path-coefficients of the mediating model.

**Dependent variable**	**Independent variable**	***B***	**95% CI**		***R*** **^2^**	***F***
			**Lower**	**Upper**		
Self-efficacy	Media use	0.053^***^	0.040	0.067	0.088	30.151^***^
Health literacy	Media use	0.200^***^	0.175	0.226	0.343	151.278^***^
	Self-efficacy	0.842^***^	0.784	0.899		

CI, confidence interval.

*** *p* < 0.001.

Model 4 of PROCESS was used, and Bootstrap sample size was 5,000. The mediating model was controlled for age, gender, residency, ethnicity, education level, current marital status, monthly personal income, occupational status, living alone, chronic disease, smoking, and alcohol.

**Table 5:** Mediating effect of self-efficacy between media use and health literacy.

	**Point estimate**	**95% CI**		**Ratio of effect**
		**Lower**	**Upper**	
Total effect	0.245^*^	0.217	0.273	
Direct effect	0.200^*^	0.175	0.226	81.63%
Indirect effect	0.045^*^	0.032	0.058	18.37%

CI, confidence interval.

* *p* < 0.05.

Model 4 of PROCESS was used, and Bootstrap sample size was 5,000.

### 3.4. Moderated mediation model

**Figure 2:**
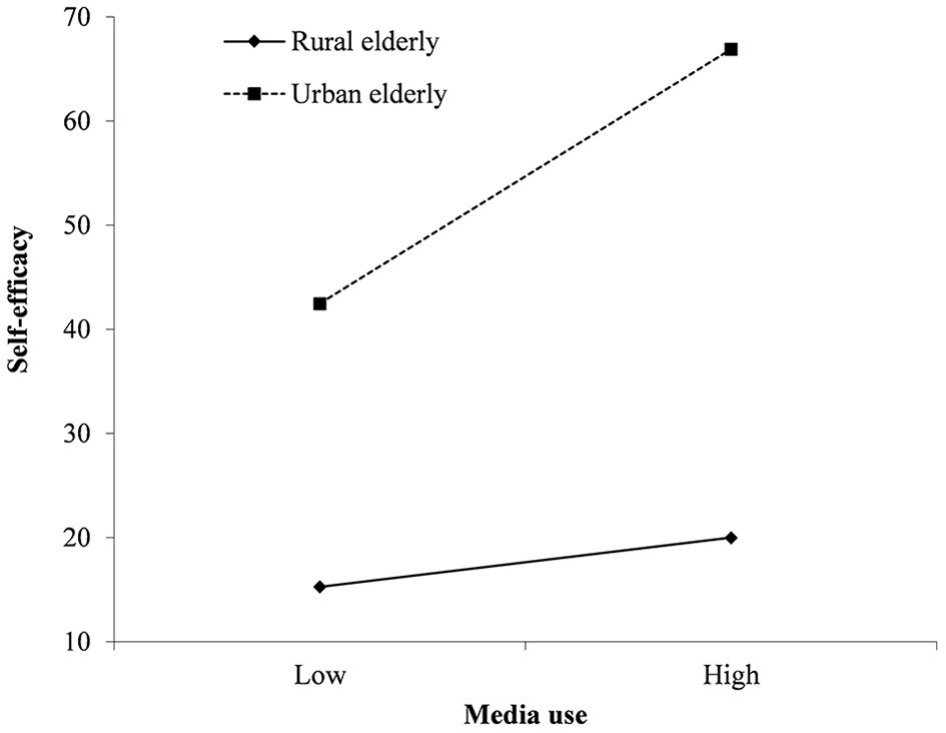
The moderating effect of urban-rural residency on the relationships between media use with self-efficacy.

**Figure 3:**
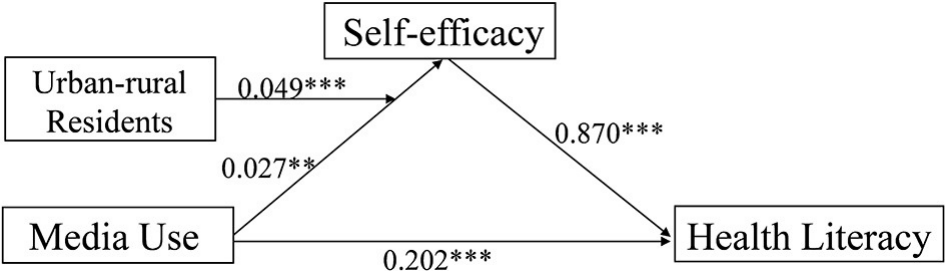
Moderated mediation model. ^**^p < 0.01; ^***^p < 0.001.

With self-efficacy as the dependent variable, media use (*B* = 0.027, 95% CI: 0.007, 0.046), urban-rural residency (*B* = 0.589, 95% CI: 0.435, 0.743), and the interaction of media use and urban-rural residency (*B* = 0.049, 95% CI: 0.024, 0.075) significantly predicted self-efficacy (Table 6). Figure 2 revealed that compared with rural elderly, the association between media use and self-efficacy was stronger for urban old people. As shown in Table 7, the indirect effect of media use on health literacy was stronger among urban aged (*B* = 0.066, 95% CI: 0.049, 0.083) compared with rural elderly (*B* = 0.023, 95% CI: 0.005, 0.041). The confidence intervals of moderated mediation index did not contain zero (*B* = 0.043, 95% CI: 0.019, 0.067), so this moderated mediation model was found to be significant (Figure 3).

**Table 6:** Path-coefficients of the moderated mediating model.

**Dependent variable**	**Independent variable**	***B***	**95% CI**		***R*** **^2^**	***F***
			**Lower**	**Upper**		
Self-efficacy	Media use	0.027^**^	0.007	0.046	0.091	29.128^***^
	Urban-rural residency	0.589^***^	0.435	0.743		
	Media use × residency	0.049^***^	0.024	0.075		
Health literacy	Media use	0.202^***^	0.176	0.228	0.332	155.036^***^
	Self-efficacy	0.870^***^	0.812	0.927		

CI, confidence interval.

** *p* < 0.01;

*** *p* < 0.001.

Model 7 of PROCESS was used, and Bootstrap sample size was 5,000. The moderated mediating model was controlled for age, gender, ethnicity, education level, current marital status, monthly personal income, occupational status, living alone, chronic disease, smoking, and alcohol.

**Table 7:** The moderating effect of urban-rural residency between media use and self-efficacy.

	**Indirect effect**	**SE**	**95% CI**	
			**Lower**	**Upper**
**Residency**				
Rural	0.023^*^	0.009	0.005	0.041
Urban	0.066^*^	0.009	0.049	0.083
Index of moderated mediation	0.043^*^	0.012	0.019	0.067

CI, confidence interval.

* *p* < 0.05.

Model 7 of PROCESS was used, and Bootstrap sample size was 5,000.

Instead of the total score of health literacy, we included the three facets of health literacy (health care, disease prevention, and health promotion) into the moderated mediation models, respectively. The results showed that the path-coefficients of the three moderated mediating models were all significant (*P* < 0.01) (Supplementary Table 2). Compared with rural elderly, urban aged people had a stronger indirect effect of media use on health care (rural: *B* = 0.007, *P* < 0.05; urban: *B* = 0.021, *P* < 0.05), disease prevention (rural: *B* = 0.008, *P* < 0.05; urban: *B* = 0.023, *P* < 0.05), and health promotion (rural: *B* = 0.008, *P* < 0.05; urban: *B* = 0.022, *P* < 0.05). The moderated mediation indexes of the three models were all found to be significant (*P* < 0.05) (Supplementary Table 3).

## 4. Discussion

In this study, we assessed the difference in media use between urban and rural elderly in China. For urban residents, health literacy was positively related to leisure and entertainment and information acquisition *via* media; for urban old people, their health literacy was associated with four media use behaviors, including leisure and entertainment, information acquisition, self-presentation, and social action. Furthermore, self-efficacy mediated the relationship between media use and health literacy, and urban-rural residency played a moderating role in the indirect influence of media use on health literacy.

The results of this study supported hypothesis 1. Urban elderly had higher frequencies than rural old people in all six types of media use behaviors, including social communication, self-presentation, social action, leisure and entertainment, information acquisition, and business transactions. That is consistent with a survey conducted in China before (39), which concluded that urban children had higher media exposure and usage than rural children. And Lariscy and his colleagues found that social media was more important for rural adolescents than urban ones (40), as there were fewer health information channels for rural residents to contact to. The urban-rural gap in media use could be mainly attributed to the difference in economic development between urban and rural areas (41). In the meantime, the health literacy score of urban older adults was also higher than that of rural aged. A systematic review had the same results (42), and they found that the urban-rural gap in health literacy was more likely to occur in developing countries. A study conducted in China also indicated that urban citizens had better health literacy than rural residents, and rural people aged 65 and above had the worst level of health literacy (43). As health literacy is related to health outcomes (14), the large health literacy gaps between urban and rural areas are likely to lead to greater health inequalities. As a result, we ought to pay more attention to the health literacy and media use performance of Chinese rural elderly and try to narrow the urban-rural gaps in the future.

Hypothesis 2 was also confirmed. Health literacy was associated with the frequency of media use, and urban and rural elderly had different types of media use behaviors that predicted their health literacy. For urban old people, leisure and entertainment *via* media and acquiring information online helped them to improve their health literacy. Mass information is provided through multiple media channels, which contain much health-related information (44). Hence, the frequency of acquiring online information could contribute to their health literacy. In addition, with the boosting of media for scientific knowledge popularization (45), the elderly can learn health-related knowledge and skills when they watch videos or listen to music. Therefore, the process of interacting with media content related to health for leisure and entertainment benefits older adults' health literacy. For the rural elderly, the same positive associations between health literacy with leisure and entertainment and acquiring information existed. Besides, their health literacy was also related to self-presentation positively and social communication negatively. Social media provides plenty of virtual platforms for users to present themselves, such as an application called Wechat in China. People would like to create or strengthen their ideal selves through posting photographs or recording personal feelings (46), to influence how others perceive them (47). It's supposed that when some rural elderly want to create good online self-images online, they would like to perform well on health-related knowledge, which could bring them more appreciated attention. As to social communication, it showed a negative relationship with the health literacy of the rural elderly. This phenomenon can be ascribed to the low health literacy of the rural resident population (42). Older adults are likely to access false or inaccurate health-related information through their communication with friends online, which may decrease their health literacy level. Nevertheless, urban citizens had more chances to create their social images among their acquaintances (48), who have a relatively high level of health literacy (43). This may weaken the importance of media self-presentation for urban seniors, and reduce their contact with wrong health knowledge. Consequently, rural elderly, rather than urban old people, had a significant association between self-presentation and social communication with health literacy. Among all participants in this study, a higher frequency of media use predicted a higher level of health literacy. Health equality can be destroyed by the digital divide, which refers to inequitable access, use, and outcomes of technology use (49). Urban residents who have higher socioeconomic status than rural people are more likely to master the ability to take use of technology (50) and improve their health further. As a result, media use is a potential factor that may reduce the growing health literacy gaps between urban and rural elderly. In the meanwhile, we found that old people with higher levels of health literacy have more frequent use of media. As better health literacy helps them to search health-related information online and to seek solutions to health problems from others *via* media. Whether there is a mutual relationship between health literacy and media use requires further exploration with longitudinal study design.

Moreover, hypothesis 3 was confirmed, which indicated that a high frequency of media use was indirectly associated with high levels of health literacy, with increasing self-efficacy as the mediator. The mediating effect of self-efficacy has been discussed in previous studies, on the relationships between external factors and health-related problems (51, 52). According to social-cognitive theory, self-efficacy determines an individual's willingness to execute specific activities, which could be influenced by self-performance and others' feedback (53). In this study, as a special behavior, media use contains personal using performance on novel things and interpersonal communication and feedback. Therefore, the self-efficacy of Chinese older adults improved through frequent media use, which further elevated the effect of learning health literacy. These findings enriched the understanding of the potential mechanism between media use and health literacy.

Furthermore, corresponding to hypothesis 4, our results suggested that the indirect effect of media use on health literacy *via* self-efficacy is moderated by urban-rural residency. That is to say, urban elderly had a stronger relationship between media use and health literacy compared with rural ones. Urban old adults performed better than rural elderly in all types of media use behaviors, which indicated that they make use of media more skillfully and deeply. It's supposed that urban elderly could obtain more enjoyment from media use in turn, which benefits their self-efficacy and then helps them to learn health-related knowledge online. In contrast, the rural elderly haven't mastered media use well, so they couldn't get enough self-satisfying and self-identity, which explains their relatively lower self-efficacy (54). As a result, with the same frequency of media use, urban elderly had higher levels of self-efficacy to improve health literacy than rural ones. Some previous studies discussed the urban-rural gap of self-efficacy (55) and the moderating effect of urban-rural residency (56), which was similar to our findings.

Several limitations of this study should be considered. Firstly, as a cross-sectional study, we could not conclude causal relationships. In the future, related longitudinal research would be conducted to establish cause-effect inference. Moreover, we used self-reported questionnaires to access the frequency of media use of participants. Therefore, reporting bias may exist, which influences the accuracy of results. We will adopt better methods to collect media use data in future work. Besides, unequal probability sampling was adopted at the community/village level, which makes samples less representative of the whole population.

Based on the above results, several essential implications could be concluded. Firstly, old people living in the countryside in China need more attention to their media use. The types of media use that impacted health literacy between urban and rural elderly were different. Therefore, health promotion programs for improving health literacy and narrowing the urban-rural gap should pay more attention to particular media use behaviors among specific groups. Secondly, health practitioners should consider self-efficacy as a short-term target in the schemes of health literacy improvement. We ought to motivate their self-efficacy first, and then make strategies to improve their health literacy, which may be more effective and scientific. Finally, this study also provides new insight to eliminate health inequality. The disparities in media use could explain the health literacy inequality, and urban-rural residency and self-efficacy play important roles in the potential mechanism of the influence of media use on health literacy.

## 5. Conclusion

This research revealed the difference in media use between urban and rural elderly in China and found that the health literacy of urban and rural elderly was associated with different types of media use behaviors. Furthermore, self-efficacy played a mediating role in the relationship between the frequency of media use and health literacy. And urban elderly had a stronger association between media use and self-efficacy in the moderated mediation model, compared with rural older adults. These findings suggested that we should make more efforts to increase the media use of rural elderly and shrink the urban-rural gap of health literacy among old people to promote health equality.

## Data availability statement

The raw data supporting the conclusions of this article will be made available by the authors, without undue reservation.

## Ethics statement

The studies involving human participants were reviewed and approved by the Ethics Committee of the Health Culture Research Center of Shaanxi [JKWH-2022-02]. The patients/participants provided their written informed consent to participate in this study.

## Author contributions

XS designed this study. YY wrote the original manuscript, prepared the analysis, and interpreted the data. YW and ZH helped with the analysis and gave essential comments on multiple versions. All authors approved the final version of the manuscript.

## References

[1] Carr CT Hayes RA. Social media: defining, developing, and divining. Atl J Commun. (2015) 23:46–65. 10.1080/15456870.2015.972282

[2] Kaplan AM Haenlein M. Users of the world, unite! The challenges and opportunities of social media. Bus Horiz. (2010) 53:59–68. 10.1016/j.bushor.2009.09.003

[3] Statista. Number of Global Social Network Users 2018-2027. (2022). Available online at: https://www.statista.com/statistics/278414/number-of-worldwide-social-network-users/ (accessed October 26, 2022).

[4] Aichner T Grünfelder M Maurer O Jegeni D. Twenty-five years of social media: a review of social media applications and definitions from 1994 to 2019. Cyberpsychol Behav Soc Networking. (2021) 24:215–22. 10.1089/cyber.2020.0134 33847527 PMC8064945

[5] Zhang K Kim K Silverstein NM Song Q Burr JA. Social media communication and loneliness among older adults: the mediating roles of social support and social contact. Gerontologist. (2021) 61:888–96. 10.1093/geront/gnaa197 33284972

[6] Center PR. Social Media Fact Sheet. (2021). Available online at: https://www.pewresearch.org/internet/fact-sheet/social-media/ (accessed October 26, 2022).

[7] Center CINI. Statistical Report on the Development of Internet in China 2021. (2021). Available online at: https://www.cnnic.net.cn/n4/2022/0401/c88-1132.html (accessed October 26, 2022)

[8] Berkman ND Davis TC McCormack L. Health literacy: what is it? J Health Commun. (2010) 15:9–19. 10.1080/10810730.2010.499985 20845189

[9] Duong TV Aringazina A Kayupova G Nurjanah f Pham TV Pham KM. Development and validation of a new short-form health literacy instrument (Hls-Sf12) for the general public in six Asian countries. Health Literacy Res Pract. (2019) 3:e91–102. 10.3928/24748307-20190225-01 31294310 PMC6607763

[10] Sørensen K Van den Broucke S Pelikan JM Fullam J Doyle G Slonska Z. Measuring health literacy in populations: illuminating the design and development process of the European Health Literacy Survey Questionnaire (Hls-Eu-Q). BMC Public Health. (2013) 13:1–10. 10.1186/1471-2458-13-948 24112855 PMC4016258

[11] Liu L Qian X Chen Z He T. Health literacy and its effect on chronic disease prevention: evidence from China's data. BMC Public Health. (2020) 20:1–14. 10.1186/s12889-020-08804-4 32410604 PMC7227325

[12] van der Gaag M Heijmans M Spoiala C Rademakers J. The importance of health literacy for self-management: a scoping review of reviews. Chronic Illn. (2022) 18:234–54. 10.1177/17423953211035472 34402309

[13] Tang C Wu X Chen X Pan B Yang X. Examining income-related inequality in health literacy and health-information seeking among urban population in China. BMC Public Health. (2019) 19:1–9. 10.1186/s12889-019-6538-2 30791882 PMC6385413

[14] Svendsen MT Bak CK Sørensen K Pelikan J Riddersholm SJ Skals RK. Associations of health literacy with socioeconomic position, health risk behavior, and health status: a large national population-based survey among danish adults. BMC Public Health. (2020) 20:1–12. 10.1186/s12889-020-08498-8 32345275 PMC7187482

[15] Yang Y Zhang B Meng H Liu D Sun M. Mediating effect of social support on the associations between health literacy, productive aging, and self-rated health among elderly Chinese adults in a newly urbanized community. Medicine. (2019) 98:e15162. 10.1097/MD.0000000000015162 31008936 PMC6494366

[16] Xu LM Xie LF Li X Wang L Gao YM. A meta-analysis of factors influencing health literacy among chinese older adults. J Public Health. (2022) 30:1889–900. 10.1007/s10389-021-01638-3

[17] Chen J Wang Y. Social media use for health purposes: systematic review. J Med Internet Res. (2021) 23:e17917. 10.2196/17917 33978589 PMC8156131

[18] Rosenbaum JE Johnson BK Deane AE. Health literacy and digital media use: assessing the health literacy skills instrument–short form and its correlates among African American college students. Digital Health. (2018) 4:2055207618770765. 10.1177/2055207618770765 29942630 PMC6016563

[19] Ozkan S Tuzun H Dikmen AU Aksakal NB Caliskan D Tasci O. The relationship between health literacy level and media used as a source of health-related information. Health Literacy Res Pract. (2021) 5:e109–17. 10.3928/24748307-20210330-01 34251938 PMC8241229

[20] Nurjanah Soenaryati S Rachmani E. Media use behavior and health literacy on high school students in Semarang. In: International Conference on Social Sciences and Humanities (SOSHUM); 2016 Apr 19-21, Kota Kinabalu, MALAYSIA (2017).

[21] Bandura A Freeman WH Lightsey R. Self-Efficacy: The Exercise of Control. New York: Springer (1999).

[22] Wu F Sheng Y. Social support network, social support, self-efficacy, health-promoting behavior and healthy aging among older adults: a pathway analysis. Arch Gerontol Geriatr. (2019) 85:103934. 10.1016/j.archger.2019.103934 31466024

[23] Huang Y Zhang J. Social media use and entrepreneurial intention: the mediating role of self-efficacy. Soc Behav Pers. (2020) 48:1–8. 10.2224/sbp.9451

[24] Mahmood QK Jafree SR Mukhtar S Fischer F. Social media use, self-efficacy, perceived threat, and preventive behavior in times of Covid-19: results of a cross-sectional study in Pakistan. Front Psychol. (2021) 12:562042. 10.3389/fpsyg.2021.562042 34220597 PMC8245845

[25] Deng Z Liu S. Understanding consumer health information-seeking behavior from the perspective of the risk perception attitude framework and social support in mobile social media websites. Int J Med Inform. (2017) 105:98–109. 10.1016/j.ijmedinf.2017.05.014 28750916

[26] Li Y Bai X Chen H. Social isolation, cognitive function, and depression among Chinese older adults: examining internet use as a predictor and a moderator. Front Public Health. (2022) 10:809713. 10.3389/fpubh.2022.809713 35359786 PMC8963936

[27] Xu XY Leung AYM Chau PH. Health literacy, self-efficacy, and associated factors among patients with diabetes. Health Literacy Res Pract. (2018) 2:e67–77. 10.3928/24748307-20180313-01 31294279 PMC6607806

[28] Zhong S Wang M Zhu Y Chen Z Huang X. Urban expansion and the urban–rural income gap: empirical evidence from China. Cities. (2022) 129:103831. 10.1016/j.cities.2022.103831

[29] Hong YA Zhou Z. A profile of ehealth behaviors in China: results from a national survey show a low of usage and significant digital divide. Front Public Health. (2018) 6:274. 10.3389/fpubh.2018.00274 30320054 PMC6168620

[30] Oh S Syn SY. Motivations for sharing information and social support in social media: a comparative analysis of F Acebook, T Witter, D Elicious, Y Ou T Ube, and F Lickr. J Assoc Inform Sci Technol. (2015) 66:2045–60. 10.1002/asi.23320

[31] Wang Y Kaierdebieke A Fan S Zhang R Huang M Li H. Study protocol: a cross-sectional study on psychology and behavior investigation of Chinese residents, Pbicr. Psychosom Med Res. (2022) 4:19. 10.53388/202219

[32] Den Hamer A Konijn EA Plaisier XS Keijer MG Krabbendam L Bushman BJ. The content-based media exposure scale (C-Me): development and validation. Comput Human Behav. (2017) 72:549–57. 10.1016/j.chb.2017.02.050

[33] Whyte W Hennessy C. Social media use within medical education: a systematic review to develop a pilot questionnaire on how social media can be best used at BSMS. MedEdPublish. (2017) 6:83. 10.15694/mep.2017.000083 PMC10885254 38406464

[34] Chen G Gully SM Eden D. Validation of a new general self-efficacy scale. Organ Res Methods. (2001) 4:62–83. 10.1177/109442810141004

[35] Naveed MA Shaukat R. Health literacy predicts Covid-19 awareness and protective behaviours of university students. Health Inform Libraries J. (2022) 39:46–58. 10.1111/hir.12404 34595814 PMC8646606

[36] Sun X Wu Y Tang J Wang F Sun X He M. Development of a Short Version of the Health Literacy Scale Short-Form: Based on Classical Test Theory and Item Response Theory. (2022). Available online at: https://chinaxiv.org/abs/202211.00133 (accessed November 21, 2022).

[37] Tasnim R Sujan MSH Islam MS Ferdous MZ Hasan MM Koly KN. Depression and anxiety among individuals with medical conditions during the Covid-19 pandemic: findings from a nationwide survey in Bangladesh. Acta Psychol. (2021) 220:103426. 10.1016/j.actpsy.2021.103426 34619554 PMC8486640

[38] Hayes AF. Introduction to Mediation, Moderation, and Conditional Process Analysis: A Regression-Based Approach. New York, NY: Guilford publications (2017).

[39] Chan K McNeal JU. Children and media in China: an urban-rural comparison study. J Consumer Mark. (2006) 23:77–86. 10.1108/07363760610655014

[40] Lariscy RW Reber BH Paek H-J. Examination of media channels and types as health information sources for adolescents: comparisons for black/white, male/female, urban/rural. J Broadcast Electron Media. (2010) 54:102–20. 10.1080/08838150903550444

[41] Reed RN Messler EC Coombs TE Quevillon RP. Social media use and the acceptability of telepsychological services in rural populations. J Rural Mental Health. (2014) 38:2. 10.1037/rmh0000007

[42] Aljassim N Ostini R. Health literacy in rural and urban populations: a systematic review. Patient Educ Couns. (2020) 103:2142–54. 10.1016/j.pec.2020.06.007 32601042

[43] Wang W Zhang Y Lin B Mei Y Ping Z Zhang Z. The urban-rural disparity in the status and risk factors of health literacy: a cross-sectional survey in central China. Int J Environ Res Public Health. (2020) 17:3848. 10.3390/ijerph17113848 32485790 PMC7312746

[44] Roberts M Callahan L O'Leary C. Social media: a path to health literacy. Inf Serv Use. (2017) 37:177–87. 10.3233/ISU-170836 28972534

[45] Liu Q Zheng Z Zheng J Chen Q Liu G Chen S. Health communication through news media during the early stage of the Covid-19 outbreak in China: digital topic modeling approach. J Med Internet Res. (2020) 22:e19118. 10.2196/19118 32302966 PMC7189789

[46] Seidman G. Self-presentation and belonging on facebook: how personality influences social media use and motivations. Pers Individ Dif. (2013) 54:402–7. 10.1016/j.paid.2012.10.009

[47] Hollenbaugh EE. Self-presentation in social media: review and research opportunities. Rev Commun Res. (2021) 9:80–98. 10.12840/ISSN.2255-4165.02731518525

[48] Sun J Lyu S. Social participation and urban-rural disparity in mental health among older adults in China. J Affect Disord. (2020) 274:399–404. 10.1016/j.jad.2020.05.091 32663969

[49] Hilton JF Barkoff L Chang O Halperin L Ratanawongsa N Sarkar U. A cross-sectional study of barriers to personal health record use among patients attending a safety-net clinic. PLoS ONE. (2012) 7:e31888. 10.1371/journal.pone.0031888 22363761 PMC3282785

[50] Cheng C Gearon E Hawkins M McPhee C Hanna L Batterham R. Digital health literacy as a predictor of awareness, engagement, and use of a national web-based personal health record: population-based survey study. J Med Internet Res. (2022) 24:e35772. 10.2196/35772 36112404 PMC9526109

[51] Magalhaes E Grych J Ferreira C Antunes C Prioste A Jongenelen I. Interpersonal violence and mental health outcomes: mediation by self-efficacy and coping. Vict Offender. (2022) 17:182–98. 10.1080/15564886.2021.1880508

[52] Choi M. Association of ehealth use, literacy, informational social support, and health-promoting behaviors: mediation of health self-efficacy. Int J Environ Res Public Health. (2020) 17:7890. 10.3390/ijerph17217890 33126469 PMC7662976

[53] Peechapol C Na-Songkhla J Sujiva S Luangsodsai A. An exploration of factors influencing self-efficacy in online learning: a systematic review. Int J Emerg Technol Learn. (2018) 13:64. 10.3991/ijet.v13i09.8351

[54] Wang YX Pei F Zhai FH Gao Q. Academic performance and peer relations among rural-to-urban migrant children in Beijing: do social identity and self-efficacy matter? Asian Soc Work Policy Rev. (2019) 13:263–73. 10.1111/aswp.12179

[55] Suton D Pfeiffer KA Eisenmann JC Yee KE Carlson JJ Feltz DL. Association of self-efficacy and fatness with physical activity in rural/urban children. J Gen Intern Med. (2012) 27:276. 10.1249/01.MSS.0000400758.42956.2430958151

[56] Lin QM Abbey C Zhang YT Wang GH Lu JK Dill SE. Association between mental health and executive dysfunction and the moderating effect of urban-rural subpopulation in general adolescents from Shangrao, China: a population-based cross-sectional study. BMJ Open. (2022) 12:e060270. 10.1136/bmjopen-2021-060270 35998954 PMC9403159

