# Supplementary material for: Associations between media use, self-efficacy, and health literacy among Chinese rural and urban elderly: A moderated mediation model
Source: Front Public Health. 2023 Mar 9;11:1104904. doi: 10.3389/fpubh.2023.1104904 (PMC10034173; doi:10.3389/fpubh.2023.1104904)
Supplement: Supplementary file 1 [file Table_1.docx]

Supplementary Material

**Supplementary Table 1.** Multiple linear regression analyses for the relations of health literacy to media use.

|  | B (95%CI) | *β* | *P*-value |
| --- | --- | --- | --- |
| **Model 1:** All participants |  |  |  |
| Health literacy | 0.280(0.249, 0.312) | 0.262 | <0.001 |
| **Model 2:** Urban elderly |  |  |  |
| Health literacy | 0.265(0.224, 0.307) | 0.250 | <0.001 |
| **Model 3:** Rural elderly |  |  |  |
| Health literacy | 0.307(0.257, 0.358) | 0.272 | <0.001 |

Note. B: non-standardized coefficients; *β*: Beta, standardized coefficients; CI: confidence interval. Multiple linear regressions were adopted, with media use as dependent variables, and health literacy as independent variables. All models were adjusted for age, gender, residency, ethnicity, education level, current marital status, monthly personal income, occupational status, living alone, chronic disease, smoking, and alcohol.

**Supplementary Table 2.** Path-coefficients of the moderated mediating models (including the three subscales of health literacy respectively).

| Dependent Variable | Independent Variable | B | 95% CI | | R^2^ | F |
| --- | --- | --- | --- | --- | --- | --- |
|  |  |  | Lower | Upper |  |  |
| **Model 1:** |  |  |  |  |  |  |
| Self-efficacy | Media use | 0.027^**^ | 0.007 | 0.046 | 0.091 | 29.128^***^ |
|  | Residency | 0.589^***^ | 0.435 | 0.743 |  |  |
|  | Media use × Residency | 0.049^***^ | 0.024 | 0.075 |  |  |
| Health care | Media use | 0.063^***^ | 0.053 | 0.072 | 0.266 | 113.115^***^ |
|  | Self-efficacy | 0.279^***^ | 0.258 | 0.301 |  |  |
|  |  |  |  |  |  |  |
| **Model 2:** |  |  |  |  |  |  |
| Self-efficacy | Media use | 0.027^**^ | 0.007 | 0.046 | 0.091 | 29.128^***^ |
|  | Residency | 0.589^***^ | 0.435 | 0.743 |  |  |
|  | Media use × Residency | 0.049^***^ | 0.024 | 0.075 |  |  |
| Disease prevention | Media use | 0.052^***^ | 0.042 | 0.061 | 0.269 | 114.583^***^ |
|  | Self-efficacy | 0.305^***^ | 0.284 | 0.327 |  |  |
|  |  |  |  |  |  |  |
| **Model 3:** |  |  |  |  |  |  |
| Self-efficacy | Media use | 0.027^**^ | 0.007 | 0.046 | 0.091 | 29.128^***^ |
|  | Residency | 0.589^***^ | 0.435 | 0.743 |  |  |
|  | Media use × Residency | 0.049^***^ | 0.024 | 0.075 |  |  |
| Health promotion | Media use | 0.088^***^ | 0.078 | 0.098 | 0.308 | 138.907^***^ |
|  | Self-efficacy | 0.285^***^ | 0.262 | 0.307 |  |  |

Note. CI: confidence interval. ** p<0.01; *** p<0.001. Model 7 of PROCESS was used, and Bootstrap sample size was 5000. All models were controlled for age, gender, ethnicity, education level, current marital status, monthly personal income, occupational status, living alone, chronic disease, smoking, and alcohol.

**Supplementary Table 3.** The moderating effect of urban-rural residency between media use and self-efficacy (including the three subscales of health literacy respectively).

|  | Indirect effect | SE | 95% CI | |
| --- | --- | --- | --- | --- |
|  |  |  | Lower | Upper |
| **Model 1:** Health care |  |  |  |  |
| Rural residency | 0.007^*^ | 0.003 | 0.002 | 0.013 |
| Urban residency | 0.021^*^ | 0.003 | 0.016 | 0.027 |
| Index of moderated mediation | 0.014^*^ | 0.004 | 0.006 | 0.021 |
|  |  |  |  |  |
| **Model 2:** Disease prevention |  |  |  |  |
| Rural residency | 0.008^*^ | 0.003 | 0.002 | 0.014 |
| Urban residency | 0.023^*^ | 0.003 | 0.017 | 0.029 |
| Index of moderated mediation | 0.015^*^ | 0.004 | 0.007 | 0.023 |
|  |  |  |  |  |
| **Model 3:** Health promotion |  |  |  |  |
| Rural residency | 0.008^*^ | 0.003 | 0.001 | 0.014 |
| Urban residency | 0.022^*^ | 0.003 | 0.016 | 0.028 |
| Index of moderated mediation | 0.014^*^ | 0.004 | 0.006 | 0.022 |

Note. CI: confidence interval. * p<0.05. Model 7 of PROCESS was used, and Bootstrap sample size was 5000.
